# Quantification of Multi-Compartment Flow with Spectral Diffusion MRI

**Published:** 2024-08-12

**Authors:** Mira M. Liu, Jonathan Dyke, Thomas Gladytz, Jonas Jasse, Ian Bolger, Sergio Calle, Swathi Pavaluri, Tanner Crews, Surya Seshan, Steven Salvatore, Isaac Stillman, Thangamani Muthukumar, Bachir Taouli, Samira Farouk, Sara Lewis, Octavia Bane

**Affiliations:** 1 BioMedical Engineering and Imaging Institute, Icahn School of Medicine at Mount Sinai, New York, NY, USA.; 2 Department of Radiology/Citigroup Biomedical Imaging Center, Weill Cornell Medicine, New York, NY, USA.; 3 Berlin Ultrahigh Field Facility (B.U.F.F.), Max Delbrück Center for Molecular Medicine in the Helmholtz Association, Berlin, Germany.; 4 Department of Diagnostic and Interventional Radiology, Medical Faculty and University Hospital Düsseldorf, Heinrich-Heine-University Düsseldorf, Düsseldorf, Germany; 5 Department of Pathology, Weill Cornell Medicine, New York, NY, USA.; 6 Department of Pathology, Icahn School of Medicine at Mount Sinai, Mount Sinai Hospital, New York, NY, USA.; 7 Department of Nephrology and Kidney Transplantation Medicine, Weill Cornell Medicine, New York, NY, USA; 8 Department of Diagnostic, Molecular and Interventional Radiology, Icahn School of Medicine at Mount Sinai, Mount Sinai Hospital, New York, NY, USA.; 9 Transplant Nephrology, Icahn School of Medicine at Mount Sinai, Mount Sinai Hospital, New York, NY, USA.

## Abstract

**Purpose:**

Estimation of multi-compartment intravoxel ‘flow’ in *fD* in ml/100g/min with multi-b-value diffusion weighted imaging and a multi-Gaussian model in the kidneys.

**Theory and Methods:**

A multi-Gaussian model of intravoxel flow using water transport time to quantify fD (ml/100g/min) is presented and simulated. Multi-compartment anisotropic DWI signal is simulated with Rician noise and SNR=50 and analyzed with a rigid bi-exponential, a rigid tri-exponential and diffusion spectrum imaging model of intravoxel incoherent motion (spectral diffusion) to study extraction of multi-compartment flow. The regularization parameter for spectral diffusion is varied to study the impact on the resulting spectrum and computation speed. The application is demonstrated in a two-center study of 54 kidney allografts with 9 b-value advanced DWI that were split by function (CKD-EPI 2021 eGFR<45ml/min/1.73m^2^) and fibrosis (Banff 2017 interstitial fibrosis and tubular atrophy score 0–6) to demonstrate multi-compartment flow of various kidney pathologies.

**Results:**

Simulation of anisotropic multi-compartment flow from spectral diffusion demonstrated strong correlation to truth for both three-compartment anisotropic diffusion ($y=1.08x+0.1,\;R^{2}=\;0.71$) and two-compartment anisotropic diffusion ($y=0.91+0.6,\;R^{2}=0.74$), outperforming rigid models in cases of variable compartment number. Use of a fixed regularization parameter set to λ=0.1 increased computation up to 208-fold and agreed with voxel-wise cross-validated regularization (concordance correlation coefficient=0.99). Spectral diffusion of renal allografts showed decreasing trend of tubular and vascular flow with higher levels of fibrosis, and significant increase in tissue parenchyma flow (f-stat=3.86, p=0.02). Tubular fD was significantly decreased in allografts with impaired function (eGFR<45ml/min/1.73m^2^)(Mann-Whitney U t-stat=−2.14, p=0.04).

**Conclusions:**

Quantitative multi-compartment intravoxel ‘flow’ can be estimated in ml/100g/min with fD from multi-Gaussian diffusion with water transport time, even with moderate anisotropy such as in kidneys. The use of spectral diffusion with a multi-Gaussian model and a fixed regularization parameter is particularly promising in organs such as the kidney with variable numbers of physiologic compartments.

## 1. INTRODUCTION

Diffusion-weighted magnetic resonance imaging (DWI MRI) is a non-invasive non-contrast measure of Brownian diffusion of water protons based on dephasing from movement of molecules between time points^1,2^. Unlike contrast or spin-labelling, DWI uses movement within a voxel and calculates the speed of intravoxel motion from signal decay. Clinical DWI assumes a single compartment of motion with one ‘apparent diffusion coefficient’ of a mono-exponential fit to the DWI decay curve. When a range of multiple b-values is used in DWI, the curve diverges from a mono-exponential into a multi-exponential^3^ suggesting multiple distinct diffusion compartments^4–6^. Intravoxel incoherent motion (IVIM) is one method of advanced DWI that splits this decay curve with a bi-exponential allowing two compartments: ‘pseudo-diffusion’ and diffusion^3–5^. Pseudo-diffusion is fast diffusion measured with low b-values (<200 mm^2^/s) sensitive to fast motion often attributed to capillary flow in microvasculature^7^, while diffusion is measured with high b-values (~200–1000 mm^2^/s) and attributed to microstructure (i.e., tissue cellularity, collagen).

However, certain organs have more than two physiologic compartments of varying distinct molecular diffusion speeds; kidneys contain tissue parenchyma, tubules, and vasculature^6,8,9^, while the brain has tissue, capillary beds, as well as cerebral spinal fluid (CSF) in ventricles and subarachnoid space^10,11^. While two-compartment IVIM shows use in the brain if CSF is removed or excluded in post-processing^10–13^, multi-compartment analysis is needed when more complex diffusion is of interest, such as kidney tubular flow. One method of doing this is by expanding the rigid IVIM bi-exponential to a rigid multi-exponential^14–16^. However, this enforces a fixed number of compartments and requires starting values that may bias fits towards false compartments for voxels with a differing number of physiologic compartments. Further, as the predominant use of diffusion MRI is in the brain, traditional IVIM pseudo-diffusion has relied on capillary geometry assumptions of the brain for quantification^7^. This restricts application to assumptions of cerebral microvasculature which are known to change between physiologic states^17^. As such, a method of fitting and quantifying simultaneous flexible multiple compartment (pseudo-)diffusive flow and transport time beyond static capillary beds is needed.

This technical note presents the theory, simulation, and application of a novel method to estimate quantitative intravoxel ‘flow’ in ml/100g/min from multi b-value DWI using a multi-Gaussian model of water transport time. It avoids capillary geometry assumptions and expands flow quantification to any distinct physiologic compartment as a function of diffusion coefficient (D), and signal fraction (f). As DWI is intravoxel, this method quantifies “local” flow within a voxel^18^, thereby allowing quantification of flow independently of delay and dispersion effects^19^ from contrast or labeling. Multi-compartment anisotropic DWI signal^20^ is simulated and fit using (1) a rigid bi-exponential (2) a rigid tri-exponential and (2) diffusion spectrum imaging of intravoxel incoherent motion (spectral diffusion) without a priori compartments or starting values^21^ and compares the calculated flow to the simulated known truth for multiple physiologic compartments. Finally, application is demonstrated in a two-center study of kidney allografts for tissue diffusion, tubular diffusion, and vascular flow, all multiple distinct diffusion compartments that would be affected by disease.

## 2. THEORY

### 2.1 Quantitative Flow and Water Transport Time from Gaussian Diffusion

For a molecule undergoing Brownian motion with diffusivity coefficient D [mm^2^/s] and an assumed net drift of zero, the probability of its location at time t is a 3D Gaussian: $p(\vec{x},t)=\frac{1}{\sqrt{{\left(2\pi \right)}^{k}{\left(2Dt\right)}^{k}}}e^{-\frac{1}{2}\sum_{i=0}^{k}\frac{x_{i}^{2}}{2Dt}\;},\;\vec{x}=\left[x_{1},x_{2},\ldots x_{i}\right]$. While mean displacement will always be 0, the variance ($\sigma^{2}=2Dt$) will increase over time, i.e. a larger proportion of the original ensemble will have travelled beyond a given boundary. The speed of this increase in variance can be used to calculate ‘water transport time’ (WTT), the time it takes for 50% of an original ensemble to have diffused out of a 1mm diameter sphere. As validated against neutron capture microspheres for the vascular compartment in the brain^22^ and previously derived in detail^23^, in brief, the 3D gaussian probability can be converted to spherical coordinates and integrated over a sphere of a diameter of 1.

(1) $$\int_{0}^{2\pi }\;\int_{0}^{\pi }\;\int_{0}^{r=0.5mm}\;\frac{1}{\sqrt{{\left(2\pi \sigma^{2}\right)}^{3}}}e^{-\frac{r^{2}}{2\sigma^{2}}r^{2}}sin\;\theta drd\theta d\phi =0.50$$

As σ is a function of time, solving Eq. 1 for σ can determine the WTT as a function of diffusion coefficient D. Integration by parts returns

$$\frac{4\pi }{{\left(2\pi \sigma^{2}\right)}^{\frac{3}{2}}}\left[-0.5\left(\frac{2\sigma^{2}}{2}e^{-\frac{0.5^{2}}{2\sigma^{2}}}\right)+\frac{\sqrt{\pi }}{4}{\left(2\sigma^{2}\right)}^{\frac{3}{2}}erf\left(\frac{0.5}{\sqrt{s}}\right)\right]=0.50$$

and the erf approximation (Eq. 7.1.27 from Abramowitz and Stegun^24^) returns:

(2) $$\frac{4\pi }{(\sqrt{0.21\pi }{)}^{3}}\int_{0}^{0.5}\;e^{-\frac{r^{2}}{0.21}}r^{2}dr=0.5021$$

In Eq. 2, $2\sigma^{2}=0.21;\;\sigma =0.32$. Therefore, with $\sigma =\sqrt{2Dt}$

(3) $$WTT=\frac{{\left(0.32\right)}^{2}}{2D}$$

WTT can be used for quantification of flow in the denominator of the central volume theory shown in Eq. 4.

(4) $$Flow=\frac{Volume}{Time}[ml/100\;g/min]$$

Volume can be calculated as $\frac{f\times f_{w}}{\rho }$ with signal fraction (f) of the compartment, water fraction $(f_{w}$) of the volume, and grams per volume (ρ)^7^. With Eq. 3 as time, this returns a quantitative measure of flow in ml/100g/min.

(5) $$\;Flow=\frac{Volume}{WTT}=\frac{f\;\times \;f_{w}}{\rho \left[\frac{g}{mL}\right]}\times \frac{2D}{{\left(.32\right)}^{2}}[s]\;=fD\times \frac{2f_{w}}{{\left(0.32\right)}^{2}\rho }\times 100\times 60[ml/100g/min]$$

If $f_{w}$ and ρ can be approximated as constant for a given organ, fD itself can be a proxy for absolute instantaneous intravoxel flow for any physiologic compartment with a known f and a known D. Therefore, Eq. 5 is a potential method to estimate intravoxel flow of any distinct diffusing compartment including structural tissue, tubules, cerebrospinal fluid, or vascular flow, while remaining independent of bolus kinetics, spin-labelling, or contrast delay and dispersion.

## 3. Methods

### 3.1 Multi-Gaussian Intravoxel Motion

If a decay curve can be split into distinct multi-exponential components, each component can be considered independently as its own Gaussian for Eq. 1 through Eq. 5, beyond just capillary perfusion^6,25^. A simulation was written (Python 3.11.4, Anaconda Inc., 2024) to demonstrate this theory in a cortical renal voxel composed of tissue (f=0.6, D=0.002mm^2^/s), tubular (f=0.3, D=0.2mm^2^/s), and vascular (f=0.1, D=0.08mm^2^/s) compartments chosen from literature healthy renal values^15,16^.

### 3.2 Spectral Diffusion of Intravoxel Motion

When the number of fluid compartments in a voxel is unknown, diffusion spectrum imaging^21^ of a multi-b-value DWI decay curve (spectral diffusion) can be used to detect distinct compartments *without* a priori assumptions of the number of compartments or starting value. In this work, the DWI decay curve was fit to an unconstrained sum of exponentials via non-negative least squares (NNLS^26^) of 300 logarithmically spaced D values^21,27–30^. This is shown in Eq. 6 for M diffusion basis vectors $D_{j}$, and N b-values $b_{i}$.

(6) $$y_{i}=\sum_{j=1}^{M}\;s_{j}e^{-b_{i}D_{j}},i=1,\;2,\ldots N$$

Minimizing the difference between Eq. 6 and the DWI decay curve outputs a spectrum of the contributions of all M exponential basis vectors. A generalized regularization term^30^ was used to smooth in the presence of noise and reduce overfitting, weighted by λ in the second term in Eq. 7.

(7) $$\chi_{r}^{2}=min\left[\sum_{i=1}^{N}\;\;{\left|\sum_{j}^{M}\;\;s_{j}e_{i}^{-b_{i}D_{j}}-y_{i}\right|}^{2}+\lambda \sum_{j=2}^{M-1}\;\;{\left|s_{j+1}-2s_{j}+s_{j-1}\right|}^{2}\right]$$

**Figure 1.**
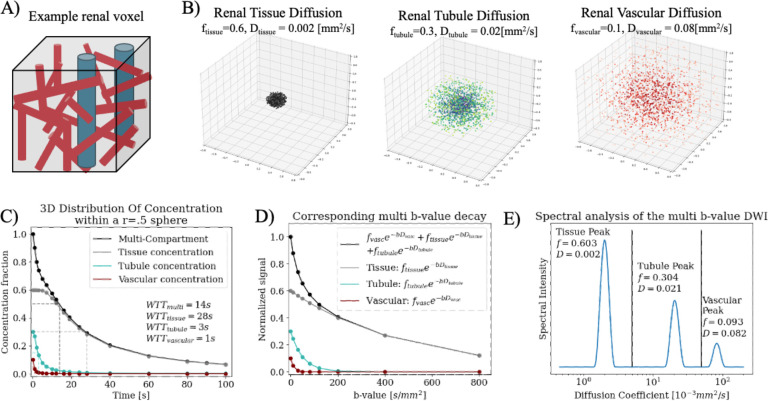
(A) Example components of a renal voxel with tissue (grey), tubules (blue), and vascular (red) components. (B) Simulated multi-gaussian diffusion over 100s within a 1mm diameter sphere. Three compartments are simulated of tissue (*f*=0.6, *D* = 0.002), renal tubules (*f*=0.3, *D*=0.02), and capillary network (*f*=0.1, *D*=0.08). (C) shows the simulated concentration of each compartment (grey, blue, red) over time as each diffuses out of the r=0.5mm sphere, with total concentration overlaid in black; the slowest diffusion is constant for the first four time points as the diffusion coefficient is so small that none have left the sphere yet. (D) shows the DWI multi b-value curve as a tri-exponential of the three compartments (black) along with corresponding individual curves from each (grey, blue, red). (E) Shows the spectrum of the simulated multi b-value curve and the fraction *f* and diffusion coefficient *D* of the three returned peaks. They are labeled as they correspond to each of the three original Gaussians.

This weighting factor λ can be selected by generalized cross validation^21,29^ for every decay curve or fixed as a constant for all analyses if SNR can be assumed as reasonably stable across curves. A lower λ allows for sharper spectral peaks while higher λ returns broader spectra and removal of small peaks due to noise. The resulting spectrum has log-normal peaks that correspond to each individual diffusion compartment; a compartment’s fraction f is the area under a spectral peak while its diffusion coefficient D is the compartment’s weighted mean diffusion coefficient. For a diffusion spectrum with amplitude $s_{i}$ for each of the $300{\;D}_{i}$ coefficients (e.g. Fig. 1E), a peak’s signal fraction was calculated as the area under the peak $f=\frac{\sum s_{k}}{\sum s_{all}}$ for the subset of indices k⊆(1,M) within a peak; the corresponding diffusion coefficient was calculated as the weighted average coefficient of the peak $D=\frac{\sum s_{k}D_{k}}{\sum s_{k}}$. With the multi-Gaussian diffusion model of intravoxel motion, each distinct peak in a diffusion spectrum represents a (pseudo-)diffusion Gaussian corresponding to a different physiologic compartment. The quantitative flow can be calculated using f and D for each compartment.

### 3.3 Influence of anisotropy on Multi-Gaussian Intravoxel Motion:

Many ‘pseudo-diffusing’ compartments, including in the kidney, are not isotropic^31–34^. A simulation of multi-compartment anisotropic diffusion was written (Python 3.11.4, Anaconda Inc., 2024) with diffusion compartments simulated as anisotropic ellipsoids. Three were created and given a diffusion coefficient D, fractional anisotropy, and signal fraction, and rotated randomly in 3D space before being measured along the global reference frame and each averaged for an approximate D. The three approximate D values were used to generate decay curves and Rician noise added for an SNR=50. Anisotropic ellipsoids and corresponding decay curves were generated and run 500 times with values chosen at random from normal distributions of literature values^21,31–33^ (Table 1a).

The 500 curves were fit with (1) a rigid bi-exponential with a standard two-step fit^5^ (2) a rigid tri-exponential and (3) with spectral diffusion. The starting values[bounds] for both rigid models were $f_{tissue}=0.7[0,1]$, $D_{tissue}=0.001[0,0.1]$, $f_{vasc}=0.1[0,1]$, $D_{vasc}=0.1[0,0.2]$, and for the tri-exponential $f_{tubule}=0.2[0,1]$, $D_{tubule}=0.01[0,0.1]$. Spectral diffusion was run with four methods: λ chosen per curve by generalized cross-validation ($\lambda_{CV}$), and with the regularization parameter fixed at $\lambda =0.1\left(\lambda_{0.1}\right)$ and $\lambda =8\left(\lambda_{8}\right)$. These parameters were chosen to cover a range of SNRs, with optimal $\lambda \approx \frac{\#bval}{SNR}$.^21^ The effects of artifacts, motion^35^, distortion, and cardiac pulsatility^36^ were not included in the simulation. Average goodness of fit, mean difference, and percent error of fit fD was calculated. Fit fD was correlated against truth ($fD_{\text{trace}}$) for the three-compartments with Spearman’s rank and linear regression. The rigid bi-exponential compartments were assigned to vasculature and tissue parenchyma and the tubule compartment set to zero. The same simulation was also run with two Gaussians to mimic a voxel with the standard two-compartment IVIM model.

### 3.4 Multi-compartment flow in renal allografts

#### 3.4.1 Two-center renal allograft study

Advanced DWI was collected in 5 healthy volunteer kidneys and 54 patient allografts using data from a prospective, IRB-approved HIPAA-compliant two-center study. Serum creatinine was collected for measurement of eGFR calculated with CKD-EPI 2021 criteria^37,38^. To compensate for single kidney filtration, eGFR≥ 45 ml/min/1.73m^2^ was considered normal/stable function, and eGFR<45 ml/min/1.73 m^2^ considered impaired function. Interstitial fibrosis and tubular atrophy (IFTA=ci+ct) scores (range, 0–6) were extracted from the clinical biopsy report scored according to the Banff 2017 classification^39^.

#### 3.4.2 MRI Protocol and post-processing

Patients underwent 3T MRI (Site 1: Skyra, Siemens Healthcare, Site 2: Prisma, Siemens Healthcare) respiratory gated 2D coronal spoiled gradient EPI DWI with denoising and motion correction and 9 b-values (b-values: 0, 10, 30, 50, 80, 120, 200, 400, 800 mm2/s; TR/TE = 1500/58ms, voxel size = 2×2×5mm3, 4-directions, 16-slices, 5mm-spacing, 3-averages). Cortical ROIs were drawn by Observer 1 (an abdominal radiologist with 14 years of experience), and spectral diffusion run on a voxel-wise basis. Spectral diffusion was run with $\lambda_{CV}$, $\lambda_{0.1}$, and $\lambda_{8}$ for; one-to-one agreement between flow from the three methods was calculated via Lin’s concordance correlation coefficient (CCC). Spectral peaks were sorted into (1) vascular, (2) tubular, and (3) tissue parenchyma diffusion and the largest peak closest to 1.8 10^−3^ mm^2^/s, a literature value between two-compartment and three-compartment rigid fits^15^, was considered the tissue parenchyma peak. Beyond that, peaks were sorted as 0.8 < tissue < 5 ≤ tubule < 50 ≤ vascular for diffusion coefficients in units of 10^−3^ mm^2^/s^8,9,15,16^. Using Eq. 5 with $f_{w}=0.80$ and an average kidney as 120g with a density of 1ρ=1g/mL ^40–42^ quantitative flow was calculated as

(8) $$Flow\;[ml/min/kidney]=fD\;\times \frac{2(0.80)}{\left(0.32^{2}\right)}\times 100\times 60\;[mL/100g/min]\times 120g/kidney\;\;=fD\times 112,500\;ml/min$$

Vascular and tubular flow using Eq. 8 was reported for the healthy volunteers and four diagnostic groups according to their kidney function and fibrosis (IFTA>0): (1) “stable allografts” - normal/stable function and no fibrosis, (2) impaired function and no fibrosis, (3) normal/stable function and fibrosis, (4) impaired function and fibrosis.

#### 3.4.3 Multi-compartment flow biomarker for kidney pathology

To examine multi-compartment flow in differing pathologic states, renal allografts were split into diagnostic classifications for function and fibrosis. Multi-parametric flow was grouped by (1) IFTA scores for allografts with normal/stable function and (2) renal function for allografts without fibrosis. Difference in multi-compartment flow between dichotomized renal function was analyzed with Mann-Whitney U-test, while difference between fibrosis levels was analyzed with one-way ANOVA, both at the p = 0.05 level. Allografts with both fibrosis and impaired function were excluded to avoid confounding trends.

## 4. RESULTS

### 4.1 Simulated multi-Gaussian IVIM

A diagram of the simulated kidney voxel with tissue, tubular, and vascular physiology (Fig. 1A) is shown with the corresponding Gaussian diffusions over time^15,16^ (Fig. 1B). The concentration of total original molecules in the voxel decreased over time (Fig. 1C, black) with each individual compartment diffusing at different rates, with three separate WTT times (Fig. 1C, red, blue, grey).

The corresponding DWI decay curve is a tri-exponential (Fig. 1D, black) sum of each individual compartment (Fig. 1D red, blue, grey). Spectral diffusion of the decay curve in 1D is shown in Fig. 1E; comparison between Fig. 1B and Fig. 1E shows the spectral peaks agree well with the three original isotropic Gaussians.

### 4.2 Influence of anisotropy on Multi-Gaussian Intravoxel Motion fD

For the three-compartment simulation, average goodness-of-fit was comparable (Table 1b). The percent error for vascular fD was smallest for the bi-exponential at the cost of no tubule fD (Table 1b). fD from spectral diffusion had the highest Spearman’s rank correlation and linear regression closest to unity (Table 1c). The bi-exponential fit showed a reduced correlation coefficient and a negative offset for fD due to the lack of a tubule compartment. For the two-compartment simulation with vascular perfusion and tissue diffusion, the rigid bi-exponential and spectral diffusion multi-compartment fD outperformed the rigid tri-exponential that was forced to fit a non-existent compartment (Table 1d).

### 4.3 Influence of spectral diffusion regularization factor λ selection

For anisotropic simulation, the multi-compartment flow calculated from spectral diffusion with $\lambda_{CV},\;\lambda_{0.1}$, or $\lambda_{8}$ showed no significant difference in correlation or linear regression to truth. Compared to $\lambda_{CV}$, a fixed λ reduced the computation time 7.4-fold for $\lambda_{0.1}$ and 4.8-fold for $\lambda_{8}$ for a three-compartment model (9.8-fold and 4.5-fold for two-compartment, respectively). $\lambda_{0.1}$ returned the greatest number of spectral peaks while $\lambda_{8}$ performed similarly to $\lambda_{CV}$ (Number of curves with 2 peaks=[487,477,477] for the two-compartment simulation, and number of curves with 3 peaks=[464,432,433] for the three-compartment simulations, with $\lambda_{0.1},\lambda_{8},\lambda_{CV}$, respectively).

### 4.3 Estimation of tubular flow in ml/min per kidney with multi-Gaussian IVIM:

**Figure 2.**
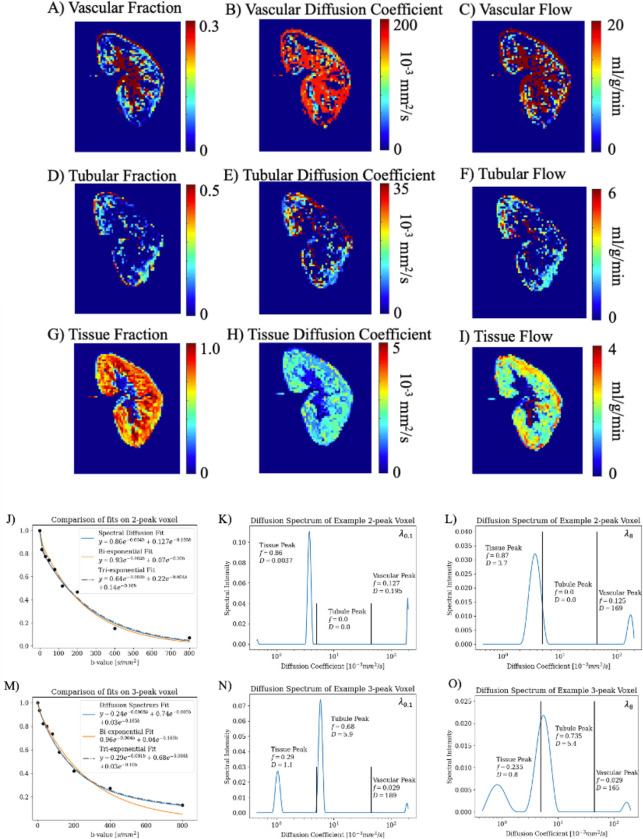
Images from a volunteer native kidney (M/47y) for (A-C) vascular spectral diffusion parameter and flow maps, (D-F) tubular spectral diffusion parameter and flow maps, and (G-I) tissue parenchyma spectral diffusion parameter and flow maps. Note the difference in scale for each compartment and parameter. (J) A voxel from the volunteer that returned two spectral peaks with the DWI decay curve and diffusion spectrum fit, bi-exponential fit, and tri-exponential fit overlaid. (K) The corresponding diffusion spectrum of (J) fit with a fixed $\lambda_{0.1}$ labeled with the fraction and diffusion coefficient, which were used to plot the diffusion spectrum fit in (J). Plotted in (L) is the diffusion spectrum of (J) with a fixed $\lambda_{8}$. (M-O) are the same as J-L for a voxel that returned three spectral peaks. A lower λ returns sharper peaks, and slightly higher weighted mean D due to a narrower peak in a log scale. Due to the difference in amplitude of peaks, with different scales for K-L and N-O, fraction is minimally changed.

Example DWI and quantitative compartment flow maps with $\lambda_{0.1}$ of a volunteer are shown in Figure 2A–I. The spectral map in Fig 2 took $t_{\lambda_{0.1}}=16s$ and $t_{\lambda_{8}}=115s$ while with voxel-wise cross-validated λ took $t_{\lambda_{CV}}=3334s$; a fixed-regularization had a 208-fold and 29-fold decrease in computation time, respectively. The DWI decay curve of two example voxels from a healthy volunteer is shown with (1) bi-exponential (2) tri-exponential and (3) spectral fits in Figure 2C–D. In Fig 2C the tri-exponential fit a false ‘tubule’ compartment with a diffusion coefficient close to the vascular compartment. Like the simulation, across all 5122 cortical allograft voxels analyzed, spectral diffusion with $\lambda_{8}$ or cross-validated returned fewer spectral peaks than $\lambda_{0.1}$. Percent 1-peak=[14.8%,18.7%,18.6%], percent 2-peak=[37.3%,42.2%,42.4%], percent 3-peak=[32.3%,34.8%,34.8%,], percent 4-peak=[4.1%,2.4%,2.3%] for $\lambda_{0.1},\;\lambda_{8}$ and $\lambda_{CV}$ respectively. However, after sorting the peaks, the resulting multi-compartment flow was not different between the three λs (CCC = 0.99, 1.00 for fD from fits with $\lambda_{0.1}$, $\lambda_{8}$ against $\lambda_{CV}$ respectively). The estimated absolute quantitative tubular flow and quantitative vascular flow for healthy volunteer kidneys and patient allografts is shown in Table 2 using fixed $\lambda_{0.1}$. While noise and variation in value is noted, but the order of magnitude and trends agree with pathology.

### 4.4 Multi-compartment flow as markers of kidney pathology

**Figure 3.**
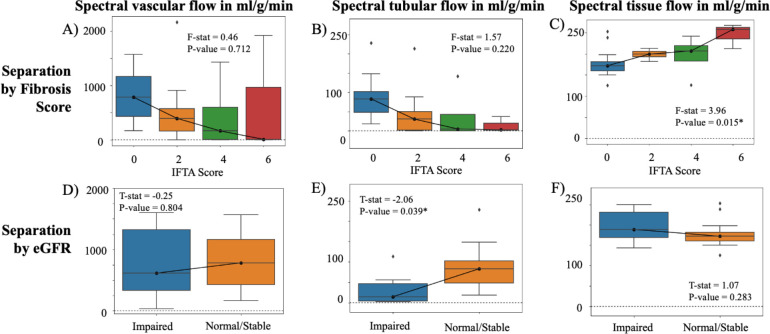
Multi-compartment flow across IFTA score (range, 0–6) for (A) vascular compartment, (B) tubular compartment, and (C) tissue parenchyma compartment. Multi-compartment flow of impaired allografts (eGFR < 45 ml/min/1.73m^2^) and normal/stable allografts (eGFR ≥ 45 ml/min/1.73m^2^) for (D) vascular compartment (E) tubular compartment, and (F) the tissue compartment. Note the different scales across the different compartments. An asterisk marks those that returned statistically significant difference at the p = 0.05 level.

Multi-compartment flow showed difference based on fibrosis (Figure 3A–C) and on function (Figure 3D–F) with spectral diffusion run with $\lambda_{0.1}$. Greater fibrosis showed significant increase in tissue parenchyma flow (F-stat = 4.18, p=0.015), which is consistent with increased collagen deposition through scarring and decreased contribution of tubular and vascular compartments (Figure 3C). Tubular fD was significantly decreased in allografts with impaired function (Mann-Whitney U-test stat = −2.06, p = 0.039; Figure 3E), which is consistent with reduced flow in kidney tubules due to reduced filtration.

## 5. DISCUSSION

Demonstrated here is a technical note of theory, simulation, and example application of measuring multi-compartment instantaneous flow in the kidneys from a multi b-value DWI acquisition. With a multi-Gaussian model of intravoxel incoherent motion and water transport time estimation, fD can represent flow of any physiologic compartment whose signal fraction and diffusion are distinct. It applies to diffusing molecules in structural tissue, tubules, cerebrospinal fluid, or vasculature independent of bolus kinetics. Spectral diffusion captured both three-compartment and two-compartment diffusion, and included tubular flow that a rigid bi-exponential from standard IVIM could not. Further, it quantified changed tissue flow in diseased parenchymal organs that exhibit restricted diffusion from pathological changes.

Spectral diffusion separated three simulated anisotropic Gaussian compartments on a similar or improved level compared to a rigid tri-exponential, and captured tubular flow that a rigid bi-exponential from standard IVIM could not. Further, it was also able to separate two anisotropic Gaussian compartments on a similar level compared to a bi-exponential and outperformed a rigid tri-exponential. As such, spectral diffusion is relevant in systems where the number of compartments cannot be assumed as constant within an organ^25,43^, even in the case of anisotropy. It could also be useful for partial volume contamination to remove unwanted signal that diffuses at a different speed than the flow of interest^44^.

Use of a fixed regularization term with $\lambda_{0.1}$ returned a 208-fold increase in computation speed of a spectral map without differing from previous^21^ voxel-wise $\lambda_{CV}$, and the resulting multi-compartment flow demonstrated statistically significant difference regarding kidney function and fibrosis score. In addition to increased computation speed, a fixed regularization term also can ensure consistency in fitting method across voxels, scans, and patients. The $\lambda_{0.1}$ returned signal trends in renal allograft advanced DWI, but for noisier signal a higher λ may be warranted/

Diffusion and multicompartment modeling MRI have shown promise in evaluation of kidney function and disease^15,16,45–51^. Simulation presented here demonstrated how the multi-Gaussian intravoxel flow model can be applied in any distinct physiologic compartment that can be reasonably approximated as isotropic and Gaussian. Further, application of the method in renal allografts showed quantification of multi-compartment flow, returning values indicative of decreased renal function and increased severity of fibrosis, both of which are expected to impact tubules and tissue^52,53^. The quantitative vascular flow agreed with the literature value for healthy volunteers^54^, and showed an expected decrease in diseased allografts.

Study of optimal b-values based on physiology of interest^55^, test-retest reliability in heterogeneous disease, and phantom validation is not included in this note. Accurate assignment of spectral peaks to physiologic compartments also requires further study and validation. Impact of drift, drag, resistance, laminar and turbulent flow in large vessels^56^ and tubules is not addressed with the model. Kurtosis seen in high b-values in highly restricted, anisotropic, complex microstructure may not be reasonably approximated as an isotropic Gaussian; this work did not use b-values above 800 s/mm^2^. The simulation did not include the effect of motion correction, T1 or T2 effects, or varying SNR per b-value. The choice of organs and clinical applications in which model-free spectral diffusion versus a fixed multi-exponential is optimal for multi-compartment flow needs further investigation. The spectral diffusion maps presented in this work may be noisier and contain more zeros than previously demonstrated rigid tri-exponential fits^15^ as it is from a single example scan without gaussian smoothing, rather than averaged across many healthy volunteers.

Multi-Gaussian IVIM has shown feasibility for quantifying multi-compartment flow in complex organs, such as the kidneys, beyond capillary perfusion beds and without the need for tagging or contrast agent. The use of spectral diffusion with this multi-Gaussian model is particularly promising in organs where voxels have variable numbers of physiologic compartments.

## Figures and Tables

**Table 1.** Anisotropy simulation of multi-Gaussian flow with bi-exponential, tri-exponential, and spectral diffusion. a) Mean and standard deviation of the normal distributions on which the simulations were based. Compartment fractions were normalized to ensure the compartments summed to one. b) Simulation results for fit accuracy of three-compartment diffusion with fractional anisotropy and Rician noise with SNR=50. The mean and standard deviation of the difference between the fit parameters and the known truth are multiplied by 10^3^ for legibility. Median percent error is reported rather than mean error to reduce outsize effects of the small known truth values in the denominator. c) Analysis of sensitivity of the fit *fD* to varying flows across all 500 three-compartment runs with Spearman’s rank correlation and linear regression against true *fD.* d) Analysis of sensitivity of the fit *fD* to varying flows across all 500 two-compartment runs with Spearman’s rank correlation and linear regression against true *fD.*

a) Distributions of simulated anisotropic Gaussians	Tissue compartment	Tubule compartment	Vascular compartment
Signal fraction (f)	0.60± 0.10	0.30±0.015	0.10±0.05
Diffusion coefficient (D)	0.0015±0.00075	0.010±0.0025	0.070±0.009
Fractional Anisotropy (FA)	0.18±0.02	0.12±0.03	0.09±0.04
**b) Compartment *fD* accuracy**	**Rigid bi-exp IVIM**	**Rigid tri-exp IVIM**	**Spectral Diffusion**
Goodness of fit r2 (mean±std)	0.986±0.007	0.997±0.003	0.996±0.01
Vascular *fD* mean difference ±std (percent error)	−0.58±2.03 (19.53%)	−1.82±2.10 (30.8%)	0.66±2.94 (29.8%)
Tubule *fD* mean difference ±std (percent error)	None	−0.44±1.40 (31.2%)	0.034±1.60 (33.7%)
Tissue *fD* mean difference ±std (percent error)	0.28±0.200 (29.8%)	−0.40±0.52 (40.4%)	−0.35±0.57 (48.2%)
**c) Comparison of fit *fD* against true three-compartment fD**	Rigid bi-exp IVIM	Rigid tri-exp IVIM	Spectral Diffusion
Spearman’s rank correlation (stat, p)	0.420, p <0.0001	0.803, p < 0.0001	0.824, p < 0.0001
Lin. Regress (linear equation, r2) of fit *fD* vs true *fD.*	*y* = 0.75*x* − 24.7 *R*^2^ = 0.64	*y* = 0.71*x* + 0.0 *R*^2^ = 0.72	*y* = 1.08*x* − 0.1 *R*^2^ = 0.71
**d) Comparison of fit *fD* against true two-compartment *fD***	**Rigid bi-exp IVIM**	**Rigid tri-exp IVIM**	**Spectral Diffusion**
Spearman’s rank correlation (stat, p)	0.99, p < 0.0001	0.70, p < 0.0001	0.92, p < 0.0001
Lin. Regress (linear equation, r2) of fit *fD* vs true *fD.*	*y* = 0.92*x* − 3.5 *R*^2^ = 0.87	*y* = 0.58 + 0.0, *R*^2^ = 0.68	*y* = 0.91 + 0.6, *R*^2^ = 0.74

**Table 2.** Quantification of vascular and tubular flow in diseased allografts. Shown are examples of calculated vascular and tubular fD in ml/min per patient using spectral diffusion parameters for diagnostic groups of fibrosis (fibrosis as IFTA>0) and function (impaired allografts having eGFR < 45 ml/min/1.73m^2^ and normal/stable allografts having eGFR ≥ 45 ml/min/1.73m^2^). The mean CKD-EPI GFR of each group is included for relevant comparison. For reference, literature standard healthy renal blood flow for is 1 liter/min total^54^.

Kidney Group	Mean vascular flow in ml/min	Mean tubular flow in ml/min	Mean ±stdev eGFR ml/min/1.72m^2^
**1. Healthy volunteer (n=5) left kidney**	605±328	122±63	>90
**2. Healthy volunteer (n=5) right kidney**	489±344	122±72	>90
**3. Stable allograft (n=13)**	807±464	82±59	57.9±10.6
**4. Allograft with impaired function and fibrosis (n=19)**	543±577	45±53	29.8±9.7
**5. Allograft with impaired function and no fibrosis (n=7)**	688±534	33±35	29.2±12.4
**6. Allograft with normal/stable function and fibrosis (n=15)**	526±661	36±56	59.1±15.2

## Data Availability

Open-source code developed to generate spectral volumes and multi-compartment flow maps described in this paper will be made publicly available on github at https://github.com/miramliu/DSI_IVIM_Maps
