# Supplementary material for: Estimation of Multi-Component Flow in the Kidney with Multi-b-value Spectral Diffusion
Source: ArXiv. 2025 Jul 30:arXiv:2408.06427v3. Originally published 2024 Aug 12. Preprint. [Version 3] (PMC11343220)
Supplement: Supplement 1 [file NIHPP2408.06427v3-supplement-1.pdf]

https://doi.org/10.1002/mrm.30644

Supporting Information S1. Influence of Spectral Diffusion Regularization Factor λ Selection
The weighting factor λ can be selected by generalized cross-validation[69,70] for every decay curve, or fixed as a constant for all analyses if signal-to-noise-ratio (SNR) is stable. A lower λ allows for sharper spectral peaks while higher λ returns broader spectra for lower SNR. Spectral diffusion was run with the regularization parameter fixed at $\lambda_{0.1} = 0.1, \lambda_2 = 2, \lambda_8 = 8$, and λ_{CV} = cross-validated from optimal $\lambda \approx \frac{\#bval}{SNR}$[13]. Multi-component fD using λ_{CV}, $\lambda_{0.1}$, or λ_8 showed no significant difference in correlation or linear regression to truth in simulation. All simulations were run on a 12 Core Apple M2 Max running MATLAB R2025a (MATLAB Engine API, 2025) and Python 3.11 (Anaconda Inc); fixed λ reduced the computation time 7.4-fold for $\lambda_{0.1}$ and 4.8-fold for λ_8 for a three-component model (9.8-fold and 4.5-fold for two-component, respectively). For fixed regularization of the spectral map in Fig.3, lower λ correlated linearly with reduced computation times ($t_\lambda(s) = 11.07\lambda + 27.3, p < 0.0001, R^2 = 0.95$). Compared to λ_{CV} fixed-regularization had a 208-fold and 29-fold decrease in computation time ($t_{\lambda_{CV}}$ =3334s, $t_{\lambda_8} = 115s$, $t_{\lambda_{0.1}} = 16s$).

For the renal allograft MRIs, with b0 SNR=50, voxels most commonly returned two-peak and three-peak spectra, with the following percentages for $\lambda_{0.1}$, λ_8 and λ_{CV} respectively: 1-peak=[14.8%,18.7%,18.6%], 2-peak=[37.3%,42.2%,42.4%], 3-peak=[32.3%,34.8%,34.8%,], 4-peak=[4.1%,2.4%,2.3%]. These were calculated as the number of peaks (i.e. signal with decreasing values on either side after smoothing, see Fig 1D. for example) in each voxel's spectrum. After sorting the peaks, multi-component flow agreed between the three λs (CCC = 0.99, 1.00 for fD from fits with $\lambda_{0.1}, \lambda_8$ against λ_{CV} respectively supporting use of $\lambda_{0.1}$ for faster computation. Visually, parameter maps did not vary noticeably between different regularization factors. Therefore, in this work, use of a fixed regularization term with $\lambda_{0.1}$ ensured consistency in fitting method across voxels, scans, and patients. As more smoothing may be needed for signal with lower SNR, $\lambda > 0.1$ may be better for processing sequences with SNR<50.

Supporting Information S2. Clinical Demographics
Table 1. Patient demographics. Patients included in the study were those enrolled from 2/2022-09/2024 who are >1-month post-transplant. Informed consent was obtained, and patients underwent a non-contrast MRI protocol within 7 days of biopsy that included advanced DWI. Exclusion criteria were age <18 years, large vessel or urinary tract complication of the kidney transplant, contra-indications to MRI, or pre-existing medical conditions including a likelihood of developing seizures or claustrophobic reactions.

**Demographics and Clinical Features**	
Biopsy Type	
Indication Biopsy	N=45
Protocol Biopsy	N=9
Sex(F/M)	21/33
Race	
Black/African American	30
White	5
Asian	6
Other/Unchecked	13
Age (years; mean±std, range)	48.8±10.5 (25-66)
Weight (kg; mean±std, range)	78.61±16.84 (44.5-118.8)
BMI (kg/m^2, mean±std, range)	27.6±5.1 (17.4-38.2)
Allograft Volume (mL, mean±std, range)	241±78 (100-573)
Living Donor	N=20;
Donor Age (years; mean±std, range)	36.41±11.33 (21-63)

https://doi.org/10.1002/mrm.30644

Deceased Donor	N=34;
KDPI	All KDPI < 85
Time since transplant (months; mean±std, range)	47.2±66.1 (1.3-252)
U-Protein (mg/24hr; mean±std, range)	159.82±284.63 (1.0-1513), 8 unknown
Donor Specific Antibodies status	42 negative, 6 positive, 6 unknown
**eGFR (CKD-EPI 2021 mL/min/1.73m^2; mean±std, range)**	44.96±19.30 (8.0-105.0)
eGFR< 45 (mL/min/1.73m^2; mean±std, range)	N=26; 28.67±9.96 (8.0-44.0)
eGFR≥ 45 (mL/min/1.73m^2; mean±std, range)	N=28; 59.01±13.49 (45.0-105.0)
**Interstitial Fibrosis/ Tubular Atrophy (IFTA)**	Total number/number with healthy eGFR
IFTA = 0	20/14
IFTA = 2	13/8
IFTA = 4	10/4
IFTA = 6	11/3
**Clinical subgroups**	
Normal/stable function and no fibrosis: eGFR≥ 45 & IFTA=0	14
Normal/stable function and fibrosis: eGFR≥ 45 & IFTA>0	15
Impaired function and no fibrosis: eGFR< 45 & IFTA=0	6
Impaired function and fibrosis: eGFR< 45 & IFTA>0	19
